# Context-Relative Norms Determine the Appropriate Type of Consent in Clinical Biobanks: Towards a Potential Solution for the Discrepancy between the General Data Protection Regulation and the European Data Protection Board on Requirements for Consent

**DOI:** 10.1007/s11948-020-00271-9

**Published:** 2020-10-13

**Authors:** R. Indrakusuma, S. Kalkman, M. J. W. Koelemay, R. Balm, D. L. Willems

**Affiliations:** 1 Amsterdam UMC, Department of Surgery, Amsterdam Cardiovascular Sciences, University of Amsterdam, Meibergdreef 9, 1105 AZ Amsterdam, the Netherlands; 2 Department of Medical Humanities, Julius Center for Health Sciences and Primary Care, University Medical Center Utrecht, Heidelberglaan 100, 3508 GA Utrecht, the Netherlands; 3 Amsterdam UMC, Department of General Practice, Section of Medical Ethics, Amsterdam Public Health Research Institute, University of Amsterdam, Meibergdreef 9, 1105 AZ Amsterdam, the Netherlands

**Keywords:** Consent, Bio bank, General data protection regulation, Article 29 working party

## Abstract

Clinical biobanks processing data of participants in the European Union (EU) fall under the scope of the General Data Protection Regulation (GDPR), which among others includes requirements for consent. These requirements are further specified by the Article 29 Working Party (WP29)—an EU advisory body currently known as the European Data Protection Board (EDPB). Unfortunately, their guidance is cause for some confusion. While the GDPR allows participants to give broad consent for research when specific research purposes are still unknown, the WP29 guidelines suggest that additional consent for specific uses should be obtained in addition to broad consent when this becomes applicable. This discrepancy elicits the question whether clinical biobanks can fail the requirement of consent if they obtain broad consent, but not a specific consent for each biomedical study. We analysed this discrepancy within the framework of contextual integrity, in order to describe the context-relative informational norms that govern information flows in clinical biobanks. However, our analysis demonstrates that there is no uniform set of norms that can be applied to all clinical biobanks. As such, neither the GDPR nor the WP29 guidance can act as a “one size fits all” approach to all clinical biobanks. Rather, differences between clinical biobanks—especially regarding the scientific aims and patient populations—make the case for context-relative norms that determine the appropriate type of consent.

## Introduction

Clinical biobanks collect human biomaterials and associated data from patients specifically for the purpose of future biomedical research (Parodi 2015). Such data are stored in a coded fashion and therefore meet the definition of personal data in the General Data Protection Regulation (GDPR) of the European Union (EU) (European Union 2016). In order to use these data, clinical biobanks need to meet one of the 6 conditions outlined by Article 6.1 which indicates when data usage can be considered lawful. These 6 conditions are: consent, contract, legal obligation, vital interest, public task or legitimate interest. Moreover, clinical biobanks process what the GDPR refers to as “special categories of personal data,” such as “data concerning health” and genetic data, which are prohibited under Article 9 unless one of the 10 conditions in Article 9.2 is satisfied. These 10 conditions are: explicit consent, employment, social security and social protection (if authorised by law), vital interest, not-for-profit body, made public by the data subject, legal claim or judicial act, reasons of substantial public interest (with a basis in law), health or social care (with a basis in law), public health (with a basis in law) and archiving, research and statistics (with a basis in law). The current approach is to use explicit consent as the legal ground for clinical biobanks under both Article 6 and Article 9, although alternative grounds could potentially be used to avoid using consent. For example, some clinical biobanks could explore the potential of using legitimate interest [Article 6.1(f)] and processing for scientific purposes where this is based on Union and Member state law [Article 9.2(j)]. As outlined by the European Data Protection Supervisor, however, no such laws currently exist and it is therefore “difficult at present, if not impossible, to view a ‘substantial public interest’ as a basis for processing sensitive data for scientific research purposes” (European Data Protection Supervisor 2020).

Where explicit consent is used as the legal basis, which will be the case for most current clinical biobanks, it must meet certain requirements, which have been described in the GDPR itself and have been further specified in guidelines of the Article 29 Working Party (WP29) (Article 29 Working Party 2018b). The WP29 guidelines on consent aim to ensure that data controllers (those that decide the means and purposes of data processing) comply with the GDPR.

WP29 was an independent advisory body of the EU and was established in the Data Protection Directive in 1995—the predecessor of the GDPR (European Union 1995). It was tasked with promoting uniform application of the Data Protection Directive in all Member States. Furthermore, WP29 was granted explicit freedom to formulate recommendations with regard to data protection, and was also expected to advise the European Commission on this matter. Although WP29 had no legal binding authority, its work which encompassed guidelines, recommendations and opinions can be considered soft law with a real impact in practice. When the GDPR came into effect, the WP29 was replaced by the European Data Protection Board (EDPB) (Article 29 Working Party 2018a). During its first plenary meeting, however, EDPB endorsed several WP29 documents, including the WP29 guidelines on consent under the scope of the GDPR (European Data Protection Board 2018). Furthermore, these guidelines have been revised as recently as 4 May 2020, although the revisions are not relevant for the discussion in this article (European Data Protection Board 2020).

Yet, there appears to be a discrepancy between the GDPR and the WP29 guidelines on consent with regard to the requirements of explicit consent for research. The basis of this discrepancy depends on the interpretation of Article 5.1.b and Recital 33 of the GDPR. Article 5.1.b is focused on limiting processing of data to its initial purpose:

Personal data shall be collected for specified, explicit and legitimate purposes and not further processed in a manner that is incompatible with those purposes; further processing for archiving purposes in the public interest, scientific or historical research purposes or statistical purposes shall, in accordance with Article 89(1), not be considered to be incompatible with the initial purposes (‘purpose limitation’).

The latter part of this article is relevant for clinical biobanks. In the context of clinical biobanks, further processing means carrying out biomedical studies with information stored in clinical biobanks. Therefore, Recital 33 explicitly recognises the challenges of specific consent for certain research purposes. It states:

It is often not possible to fully identify the purpose of personal data processing for scientific research purposes at the time of data collection. Therefore, data subjects should be allowed to give their consent to certain areas of scientific research when in keeping with recognised ethical standards for scientific research. Data subjects should have the opportunity to give their consent only to certain areas of research or parts of research projects to the extent allowed by the intended purpose.

Based on Article 5.1.b and Recital 33, it appears that clinical biobanks can fall under these exemptions for specific consent. However, the WP29 guidelines give a stricter interpretation of the GDPR in relation to research, as it states:

First, it should be noted that Recital 33 does not disapply the obligations with regard to the requirement of specific consent. This means that, in principle, scientific research projects can only include personal data on the basis of consent if they have a well-described purpose. For the cases where purposes for data processing within a scientific research project cannot be specified at the outset, Recital 33 allows as an exception that the purpose may be described at a more general level […]. When regarded as a whole, the GDPR cannot be interpreted to allow for a controller to navigate around the key principle of specifying purposes for which consent of the data subject is asked. When research purposes cannot be fully specified, a controller must seek other ways to ensure the essence of the consent requirements are served best, for example, to allow data subjects to consent for a research purpose in more general terms, and for specific stages of a research project that are already known to take place at the outset. As the research advances, consent for subsequent steps in the project can be obtained before that next stage begins.

This discrepancy between the WP29 guidelines and the GDPR is problematic because it creates uncertainty for how clinical biobanks should proceed. Two diverging scenarios appear to be possible, one where a broad consent could be sufficient for clinical biobanks (GDPR scenario), and another where consent is advised to be gained for each subsequent stage in the project, which in the context of clinical biobanks would be a biomedical study (WP29 scenario). Due to this discrepancy, the WP29 guidelines have been criticised by international stakeholders in the field of biobanking, such as Biobanking and BioMolecular resources Research Infrastructure—European Research Infrastructure Consortium (BBMRI–ERIC), who submitted comments during a public consultation (Meijer et al. 2018). BBMRI-ERIC is the largest research infrastructure in Europe and consists out of 19 members; Austria, Belgium, Czech Republic, Estonia, Finland, France, Germany, Greece, Italy, Latvia, Malta, the Netherlands, Norway, Poland, Sweden, and the United Kingdom. Collectively, they argue that the GDPR is meant to facilitate research, and that patients therefore “should not be deprived of the option to give their broad consent.” In addition, they are concerned that “the requirement of a granular re-consent system may result in a selection bias” due to consent fatigue. Such consent fatigue could limit the scientific output of any clinical biobank. More importantly, they hold the position that (re-)use of data is an ethical requirement by itself, as this prevents unnecessary repetition of biomedical studies. Similar comments were submitted by the Secretary’s Advisory Committee on Human Research Protections (SACHRP) of the United States of America (USA). They observed that the draft WP29 guidelines “could seriously restrict the availability of broad consent to future research as a basis for processing personal data under the GDPR” and that these draft guidelines are confusing with regard to Recital 33 as “the guidelines could therefore be read to suggest that providing data subjects a description of the general areas of future research […] might not satisfy the GDPR’s requirement that consent be specific.” (Secretary's Advisory Committee on Human Research Protections 2018).

Despite numerous comments, this discrepancy persists in the current version of the WP29 guidelines (European Data Protection Board 2020). If clinical biobanks exclusively follow the WP29 scenario, scarce research resources and time are spent on obtaining consent, instead of being used towards research itself. In some cases, patients may even become lost to follow-up, resulting in a permanent loss of opportunity to ask for their consent which makes storing their biomaterials and data meaningless. This has the consequence of limiting the value of biomaterials and data if these are only kept for storage in clinical biobanks. Moreover, not using the already collected data and biomaterials is also unethical because it wastes the efforts patients undertook to donate. With the prospect of practical and ethical complexities arising from adherence to the WP29 guidelines, a straightforward solution would seem to simply follow the GDPR.

The WP29 guidelines, however, do make an ethically significant point, despite the fact that their divergent practical guidance causes confusion. One of the key principles in research ethics is that subjects are required to provide their informed consent for research (World Medical Association 2013). Traditionally, informed consent is considered meaningful only if subjects are informed about the specifics of the research study. Even if there are practical difficulties with obtaining specific consent from individuals at the time of recruitment, as is the case in biobank research, a one-off type of general consent may be inadequate to respect autonomy and protect individuals’ from harm (Kaye et al. 2015). As such, the WP29 guidelines seem to warn against the waning importance of researchers’ obligation to obtain truly meaningful informed consent. Ultimately, a tension appears between the GDPR as a risk for under regulation, on the one hand, and the WP29 guidelines as a risk for overregulation, on the other.

In an attempt to reconcile this ethical tension and foster responsible research, we provide an account of contextual integrity applied to the case of clinical biobanks. Contextual integrity is a theory of privacy in context and provides us with the appropriate analytical framework to address the discrepancy between the GDPR and the WP29 guidelines (Nissenbaum 2010). A theory of privacy is justified because the GDPR is concerned with *protection of personal data*, a fundamental right established in Article 8 of the Charter of Fundamental Rights of the EU (European Union 2012). Furthermore, the GDPR is explicitly focused on the *free movement* of personal data, and it additionally notes in Recital 4 that “the processing of personal data should be designed to serve mankind. The right to the protection of personal data is not an absolute right; it must be considered in relation to its function in society and be balanced against other fundamental rights.” In other words, we are concerned with the balance between privacy and its relation to other rights in diverse societal contexts. In addition, contextual integrity deals with information *flows*, and the *contexts* in which these flows take place. It is thus well-equipped to tackle our research question for *specifically* clinical biobanks. This is in contrast to classical privacy theories that approach privacy from a perspective of “control” or “access” to one’s personal data (Rössler 2005). In these classical privacy theories, a lack of control of, or unauthorised access to, one’s personal data can already be regarded as a privacy violation irrespective of the context in which this takes place.

## Contextual Integrity

The framework of contextual integrity proposes contextual integrity as a benchmark for privacy (Nissenbaum 2010). The underlying principle of contextual integrity is that different values are at stake in different human contexts. As a result, each context comes with its own norms how information should be shared (information flows) within and between contexts. Such norms can either be explicitly (e.g. in the form of codified laws) or implicitly (e.g. as social customs) present. These norms are referred to as context-relative informational norms. A frequently given example is the difference in the amount and type of information someone is willing to share with their physician versus their colleague. Contextual integrity is concerned with whether the sharing of information is *appropriate* for the context in which it is shared taking into account the context-relative informational norms. If sharing of information breaches a context-relative informational norm, this sharing then constitutes a violation of contextual integrity and therefore privacy.

Thus, before an information practice can be said to violate privacy, it is necessary to understand the context-relative informational norms, by describing four parameters that constitute a context-relative informational norm. These parameters are: the context and its values (or goals or purposes), the people involved (actors), the type of data (attributes) and the principles that govern information flow (transmission principles). Subsequently, an information practice can then be judged on its appropriateness in comparison to these norms.

## Context-Relative Informational Norms for Clinical Biobanks

### Contexts and their Values

Clinical biobanks take place within the broader context of healthcare, usually within the context of academic hospitals. Predominant values (or goals or purposes) have been codified through globally recognised and supported declarations of the World Medical Association. Within the context of healthcare, the primary values can be learned from the Declaration of Geneva, which is also known as the physician’s pledge (World Medical Association 2017a). This declaration states that physicians are firstly concerned with the health and well-being of patients. Other values formulated in this declaration are the respect for autonomy and dignity of patients, and the confidentiality of information that patients share with their physicians. Interestingly, the declaration further prescribes that physicians are to share their medical knowledge for the benefit of patients and the advancement of healthcare.

In the context of clinical biobanks in academic hospitals, two additional declarations of the World Medical Association are relevant, the Declaration of Helsinki (medical research involving human subjects) and the Declaration of Taipei (research on health databases, big data and biobanks). The Declaration of Helsinki underlines the importance of medical progress through the conduct of biomedical research, which includes studies with patients (World Medical Association 2013). However, biomedical research can never prevail over the rights and interests of patients, such as their right to privacy. The Declaration of Taipei covers the collection, storage and use of human biomaterials and associated data beyond the individual care of patients in healthcare databases and biobanks (World Medical Association 2017b). It states that consent to a biobank can only be considered if a potential participant is adequately informed, among others about the purpose, risks and burden of participation and the rules of access to the biobank. Taken together, the three declarations demonstrate the importance of the goal to advance medical knowledge through research, while protecting patients’ rights and interests.

In addition to these declarations, clinical biobanks can represent their own contexts with goals (or scientific aims). This is because clinical biobanks are often centred around a specific disease and its corresponding patient population. Consequently, goals may differ between groups of patients as they face different health challenges, which influences what may be appropriate for research. For example, research regarding patients with incurable diseases is focused on finding any treatment irrespective of associated treatment harms, where a higher patient burden and invasive study procedures may be more easily justified. By contrast, patient burden should be kept to a minimum for patients with asymptomatic diseases, as it is usually the aim to minimise harm and maximise benefit. In other cases, research is focused on patients in the emergency care setting, where patients are acutely ill and who may or may not even be conscious. These varying contexts may therefore also inform the other parameters.

### Actors

The framework of contextual integrity identifies 3 types of actors: those whom the data concern (information subjects), those who send the data (senders) and those that receive it (recipients). Within the context of clinical biobanks, participating patients are the majority of information subjects, although data can also be collected about physicians and their care, or about relatives when genetic data is collected. However, we will focus on participating patients as the sole information subjects in the current analysis.

Participants are also senders of information because they donate biomaterials and data. The primary recipients are the initiating and coordinating research team (in GDRP terms: data controllers) and supportive collaborators (in GDPR terms: data processors). The initiating research team is often led by medical specialists who are also involved with the care of participants. Frequent supportive collaborators are the biochemical laboratory for the processing of the biomaterials, the biobank department itself for storing the biomaterials and most likely another party that stores the associated data. These supportive collaborators have (temporary) access to the biomaterials and data, but only to process it as agreed upon not for any independent or secondary purposes. These actors described thus far are an entrenched norm for clinical biobanks.

Additionally, clinical biobanks also have some form of a data sharing policy in order maximise the number of biomedical studies carried out, which creates secondary senders (the clinical biobank) and recipients (those carrying out the biomedical study). Such data sharing policies relate to two parameters of context-relative informational norms, namely actors and transmission principles. Although the parameter actors describes with *whom* data is shared with, the parameter transmission principles is where any existing norms of such data sharing are described. In the respective section later on, we will describe that there is such a norm, while we will now discuss whether there are any norms regarding whom the secondary recipients are. To our knowledge, however, there are no entrenched consensus-based norms with regard to secondary recipients. Instead, we will discuss several categories of secondary recipients relevant to our context. An important category is that of external researchers, divided in those located within and outside the EU—a relevant distinction for the GDPR. This category of secondary recipients appears to be empirically supported, for example by a survey of 308 biobank participants (recruited via a broad consent model) in the USA, who expressed that sharing their biomaterials and data with other *domestic* researchers was “extremely acceptable” (Warner et al. 2018). That survey also showed that sharing with *foreign* researchers was considered to be “moderately acceptable.” By contrast, a survey of participants in the European Diabetes Research on Patient Stratification (DIRECT) study found that 86.8% also supported sharing with global researchers, in addition to European researchers (Shah et al. 2018). Although not a biobank in name, the DIRECT study collected biomaterials and associated data from participants, set up a research database for future use and used a broad consent model and is therefore comparable to clinical biobanks.

Another category is that of commercial parties in the case of a public–private collaboration for research. A European example is the aim of Biobank Norway to contribute to drug development through collaborations with industry (Biobank Norway 2018). However, survey data shows that this category is much more contested than domestic external researchers. The previous survey by Warner et al. showed that such sharing was considered to be only “moderately acceptable”. Similar results have been observed among DIRECT participants, as only 56.5% of participants supported sharing with commercial parties (Shah et al. 2018). Moreover, data sharing with commercial parties should ideally be further characterised as either commercial or non-profit, even if this distinction can sometimes be difficult to make. Overall, these surveys show that the type of secondary recipient matters to participants, and that differing views are prevalent. This presents us with an interesting situation: although we will later on describe a norm for data sharing with secondary recipients, we cannot describe norms for with *whom* data is shared.

### Attributes

Clinical biobanks store personal data in the form of biomaterials and associated health-related data, which are considered to be a special category of personal data under the GDPR. As such, these data can only be collected when there is explicit consent for one or more specific purposes Article 9.2. Biomaterials can consist of many different types, ranging from blood samples to genetic data. Associated data is usually generated through regular care, but it is often augmented with data from study questionnaires and/or study examinations. Due to the broad scientific aims of clinical biobanks, it is likely that the associated data covers a wide range of themes. This is not to say that there are no boundaries to what is collected. The characteristics and aims of any single clinical biobank usually provides clear boundaries to what is collected. For example, it is relevant to collect genetic data for a clinical biobank that wants to assess genetic factors of a certain disease. Similarly, associated data should only collected when it is regarded as relevant to scientific aims. This carries the implicit and explicit limitation that data unrelated to the disease or to the scientific aims are inappropriate to collect. The entrenched norm is that clinical biobanks are to scientifically motivate why certain associated data and/or biomaterials are collected, which is specified in a biobank protocol. Such scientific rationale is then usually assessed by a medical-ethical committee that has the authorising power before clinical biobanks are started.

### Transmission Principles

Transmission principles are the fourth and final parameter that complete context-relative informational norms. These principles dictate the conditions of all information flows within a given context. For example, is information shared in a back and forth exchange, or is information only shared from one person to another (reciprocity)? And if information is shared, is it supposed to remain confidential or is it allowed to be shared even further to other recipients?

In clinical biobanks, patients voluntarily consent to the donation of their biomaterials and data without receiving information in return from the parties involved with managing the clinical biobank, it is therefore a unidirectional exchange of information. Participants also have the explicit freedom to refuse donation even after consenting to participation, for example not to fill in surveys or to refuse donation of a blood sample. Furthermore, they have the option of quitting their participation in the clinical biobank altogether, leading to the deletion of their clinical data and biomaterials in most clinical biobanks. Confidentiality is explicitly recognised throughout the processing, storing and eventual analyses of the information: information is saved in a coded fashion, it is protected according to the relevant standards and regulations and access to the identifying key file is limited to a select few. These aforementioned transmission principles are the entrenched norms, and are ideally formalised in a governance structure, which is one of several ethical principles highlighted by the Council for International Organizations of Medical Sciences (CIOMS) in the International Ethical Guidelines for Health-related Research Involving Humans. Further examples are whether and/or how unexpected findings should be disclosed, what method is used to maintain confidentiality and what the procedure is when a participants wants to quit (Council for International Organizations of Medical Sciences (CIOMS) 2016). Ethical governance that goes beyond legal norms ties research to responsible research conduct and helps to increase trustworthiness and, ultimately, public trust in data research (Carter et al. 2015).

A relatively new, yet widely supported norm is that clinical biobanks should allow data sharing in order to achieve its original aim: to facilitate biomedical research in the pursuit of medical progress. Moreover, this norm of data sharing from clinical biobanks is also explicitly recognised in EU law, with its decision to create BBMRI-ERIC (European Union 2013). One of its tasks is to make biomaterials and data from affiliated biobanks available to researchers, for the purpose of facilitating high-quality research, as outlined in Article 18. It has therefore created an access policy for affiliated biobanks, that describes the ethical principles and procedures regarding data sharing (BBMRI-ERIC 2017). Finally, this norm is also supported by biobank and study participants themselves (Warner et al. 2018; Shah et al. 2018). However, this sharing takes place under additional transmission principles. In particular, data sharing with secondary recipients is solely done for the purpose of an approved study protocol, has medical-ethical approval where necessary, is governed by a legal agreement and is limited to the secondary recipients it cannot be shared by the secondary recipient itself.

Although we described earlier that there is a transmission principle of voluntary consent, debate is still ongoing regarding the appropriate type of consent. In fact, several types of consent can be considered for clinical biobanks, as opposed to either broad or specific consent. Rothstein et al. lists: blanket consent, broad consent, specific consent, dynamic consent and tiered consent (Rothstein et al. 2016). These types of consents differ with respect to what role the participant retains, if any, during their participation. Blanket consent refers to the practice of a single consent moment, where a participant consents to all future research uses of their biomaterials and associated data. Similarly, broad consent entails a scenario in which participants consent once to the research enterprise of clinical biobanks, i.e. the collection and future use of their data, yet a crucial difference with blanket consent is that there is ethical oversight from medical-ethical committees to ensure that future use aligns within the scope of the clinical biobank (Rothstein et al. 2016). Conversely, specific consent requires that participants give separate consent for new biomedical studies that use their data. Finally, dynamic and tiered consent can be seen as some sort of middle ground and have been proposed as a better suited alternative to both broad and specific consent. Dynamic consent aims to periodically inform patients about future biomedical studies with their data, and seeks to give patients more control: either through the ability of opt-out to specific biomedical studies or through (general) opt-ins to certain types of biomedical studies. Dynamic consent is therefore an active process that is unique to each biobank and which requires continuous patient involvement. Consequently, it also carries a higher participant burden which may not be appreciated by all participants. Tiered consent refers to the initial consent procedure where patients are simultaneously offered an additional set of options to which they can consent (or not). For example, tiered consent has been used in biobanks that study a larger disease spectrum, so that patients can choose for which diseases their data may be studied. These different types of consent reflect the difficulty of the concept of informed consent for clinical biobanks, as practically most of its future uses are unspecified at the time of recruiting patients. Conclusively, there simply is no norm with regard to type of consent within the context of clinical biobanks (Caulfield and Murdoch 2017). Perhaps the divergence between the GDPR and the WP29 guidelines is rooted in this lack of consensus.

## From Uniformity to Diversity of Norms

Our application of the framework of contextual integrity demonstrates that there are no uniform norms to favour either the GDPR, or the WP29 guidelines, when it comes to the appropriate type of consent. Instead, different types of clinical biobanks require different sets of context-relative informational norms. The underlying rationale for such an approach is based on the diversity of the contexts and contextual goals (i.e. broad scientific aims of each clinical biobank), the type of information subjects (i.e. type of patients) involved and the type of information to be collected. Although it may sound like a contradiction, broad scientific aims can be specific in the sense that they matter *specifically* for the patient population in question yet they are broad in the sense that the aims do not include specific details. Different sets of contextual-relative informational norms may govern information flows for these patient populations, especially regarding the two parameters for which we were unable to describe general norms earlier—type of consent and type of secondary recipients. A recommendation would be to tailor at least these two norms to the type of clinical biobank. In the case of a clinical biobank about rare diseases, norms for broad consent and international data sharing may be justified to overcome the scientific challenge of small sample sizes. By contrast, a clinical biobank comprising potentially vulnerable patients with mental health illness could opt for specific consent when both a study protocol and the secondary recipient become known. In other, more straightforward cases, a tiered consent form could be chosen: broad consent with additional consent options for specific parts of the clinical biobanks, e.g. for collecting and analysing genetic data or for sharing with external researchers.

The parameters of the framework of contextual integrity could be used as a template, with which researchers can propose to which norms their future clinical biobank will adhere. This encourages researchers to provide an ethical justification for their norms of choice. As such, it becomes less about uniformity of norms but much more about the way in which diverse norms can be accounted for. This approach enables a type of governance that can be taken up by existing oversight committees, for example by the medical-ethical and/or biobank committee. The role of a medical-ethical and/or biobank committee would be to assess whether sufficient ethical justification exists for the proposed norms and whether they are appropriate for the specific context. Formalising and documenting norms and their justification also enables transparency and helps to avoid arbitrariness regarding the approval procedure. Moreover, the medical-ethical and/or biobank committees will have to use the authorised norms in order to assess whether the clinical biobank remains within scope. This pro-active, tailored approached recognises the fact that there is no “one size fits all” solution for clinical biobanks, and circumvents the false dichotomy between the GDPR and WP29 scenario.

## Conclusion

Although clinical biobanks are united by the overarching goal of enabling future biomedical research, there is no uniform set of context-relative informational norms that could govern information flows for all clinical biobanks. Consequently, the discussion needs to progress beyond choosing exclusively for either the GDPR or the WP29 guidelines. The diversity among clinical biobanks, specifically with regard to the contextual goals and patient populations, warrants context-relative norms that inform the appropriate type of consent.
